# Systemic Cytokine Expression in Diabetes Is Associated with Prolonged Gastrointestinal Transit Times and Cardinal Gastroparesis Symptoms

**DOI:** 10.3390/biomedicines11041027

**Published:** 2023-03-27

**Authors:** Tina Okdahl, Anne-Marie Wegeberg, Anne Birthe Helweg Jensen, Sarah Thorius Jensen, Helene Riis Pontoppidan Andersen, Joachim Størling, Birgitte Brock, Christina Brock

**Affiliations:** 1 Mech-Sense, Department of Gastroenterology and Hepatology, Aalborg University Hospital, 9000 Aalborg, Denmark; 2 Thisted Research Unit, Aalborg University Hospital Thisted, 7700 Thisted, Denmark; 3 Steno Diabetes Center Copenhagen, 2730 Herlev, Denmark; 4 Department of Biomedical Sciences, University of Copenhagen, 1165 Copenhagen, Denmark; 5 Department of Clinical Medicine, Aalborg University, 9000 Aalborg, Denmark; 6 Steno Diabetes Center Northern Jutland, 9000 Aalborg, Denmark

**Keywords:** enteric nervous system, diabetes, gastroenteropathy, inflammation, cytokine, wireless motility capsule

## Abstract

Gastroenteropathy is a common complication in diabetes associated with damages to the enteric nervous system. Systemic low-grade inflammation facilitates neurotoxicity, and associations with peripheral and autonomic neuropathy have been reported. However, less is known of associations with gastroenteropathy. To explore the area cross-sectionally, we included individuals with diabetes (type 1: 56, type 2: 100) and 21 healthy controls. Serum levels of interleukin (IL)-6, IL-8, IL-10, tumour necrosis factor (TNF)-α, and interferon (IFN)-γ were measured by multiplex technology. Segmental gastrointestinal transit times were assessed by wireless motility capsule investigations. Symptoms of gastroparesis were rated on Gastroparesis Cardinal Symptom Index questionnaires. Compared to healthy, levels of TNF-α were decreased in type 1 diabetes and increased in type 2 diabetes, while colonic transit time was increased (all *p* < 0.05). In diabetes, associations between IL-8 and prolonged gastric emptying (odds ratio (OR) 1.07, *p* = 0.027) and between IL-10 and prolonged colonic transit (OR 29.99, *p* = 0.013) were seen. Inverse correlations between IL-6 and nausea/vomiting (rho = −0.19, *p* = 0.026) and bloating (rho = −0.29; *p* < 0.001) were found. These findings indicate a plausible interaction between inflammation and the enteric nervous system in diabetes, which raises the question of whether anti-inflammatory strategies could be applied in management of diabetic gastroenteropathy.

## 1. Introduction

Individuals with diabetes mellitus are at increased risk of developing gastroenteropathy, typically associated with symptoms such as nausea, early satiety, bloating, and constipation [1]. The condition seems to be pan-enteric even though much focus has traditionally been put on gastroparesis, being the most common gastrointestinal complication with a hazard ratio of 33 in type 1 diabetes and 7.5 in type 2 diabetes compared to healthy [2]. Gastrointestinal dysfunction is influenced by disturbances in the timely and efficient motility patterns regulated by the enteric nervous system, which is a part of the autonomic regulation of homeostasis. The enteric nervous system is located in the submucosal and myenteric plexus throughout the wall of the gastrointestinal tract and is particularly vulnerable to glycaemic fluctuations [1,3]. Exact diagnosis of diabetic gastroenteropathy can be done by evaluating submucosal biopsies but is otherwise complicated by the lack of known non-invasive methods for direct assessment of the enteric nervous system. It is, however, generally accepted that gastrointestinal transit times can be used as a proxy for myogenic regulation. Indeed, previous studies have shown prolongation of transit times in all gastrointestinal segments of the diabetic gut compared to those of healthy [4,5]. 

The pathophysiology of diabetic gastroenteropathy is complex but involves structural changes to the wall of the gastrointestinal tract, including deterioration of the enteric nervous system, loss of enteric glial cells, myopathy of smooth muscle cells, and angiopathy [1]. In patients with ulcerative colitis, an association between increased levels of cytokines and prolonged orocecal transit has been shown [6]. Individuals with diabetes have systemic low-grade inflammation caused by metabolic changes leading to activation of pro-inflammatory pathways and oxidative stress. Moreover, differences in inflammatory biomarkers between males and females have been reported [7]. Evidence suggests that heightened systemic inflammation is associated with microvascular complications, including retinopathy, nephropathy, and neuropathy [8]. Moreover, systemic low-grade inflammation is associated with objective measures of commonly diagnosed complications such as distal symmetrical polyneuropathy and diabetic autonomic neuropathy [9,10]. Consequently, circulating biomarkers may reflect local neuroinflammation in diabetes [11,12,13], and it is plausible that systemic inflammation also impairs the enteric nervous system, thereby deteriorating motility in diabetes, causing gastroenteropathy.

For this study, we hypothesized that elevated systemic levels of inflammatory markers are associated with diabetic gastroenteropathy. Thus, our aim was to explore whether individuals with diabetes and normal or prolonged segmental gastrointestinal transit times exhibit differences in the systemic levels of the inflammatory cytokines (interleukin (IL)-6, IL-8, IL-10, interferon (IFN)-γ, and tumour necrosis factor (TNF)-α). Secondly, the aim was to investigate correlations between inflammatory cytokines and subjective symptoms of gastroparesis.

## 2. Materials and Methods

### 2.1. Study Population

In this cross-sectional study, we included 156 individuals with type 1 (*n* = 56) or type 2 (*n* = 100) diabetes (HbA1c ≥ 6.5%) verified for a minimum of one year. All participants were adults (age above 18 years) and of Northern European descent. Recruitment took place at the Department of Endocrinology at Aalborg University Hospital, Denmark, in connection with annual diabetes visits. Diabetic complications were assessed as described in [8]. The study was conducted in accordance with the Declaration of Helsinki. The North Denmark Region Committee on Health Research Ethics approved the study protocol and amendments (N-20170045). All participants gave their signed informed consent before participating in the study. Additionally, a cohort of healthy controls (*n* = 21) was included (N-20090008).

### 2.2. Inflammatory Cytokines

Fasting blood samples were collected from the cubital vein, and serum was isolated by centrifugation and stored at −80 °C until all samples were collected. Inflammatory biomarkers (IL-6, IL-8, IL-10, TNF-α, IFN-γ, and C-reactive protein (CRP)) were analysed at Steno Diabetes Center Copenhagen, Denmark using the V-PLEX Neuroinflammation Panel 1 Human Kit (Meso Scale Diagnostics, LLC, Cat. No. K15210D-1). All samples were analysed in duplicates. Samples were excluded if the coefficient of variance was above 30% between duplicate measurements (IL-6: *n* = 1; TNF-α: *n* = 1). Samples with concentrations below the detection limit were allocated a value equal to the detection limit divided by the square root of two (IL-6: *n* = 1) [14]. Samples from participants with CRP levels >10 mg/L were excluded to account for elevated levels in inflammatory biomarkers, which were not related to long-term diabetes, but likely due to recent or present infection. 

### 2.3. Gastrointestinal Transit Times

Gastrointestinal transit times were investigated using the wireless motility capsule (SmartPill; Given Imaging, Israel) as described in detail elsewhere [15]. Following an overnight fast, participants consumed a standardized test meal (SmartBar, Medtronic, Watford, UK; 260-kcal, composed of 3% fat, 21% protein, and 75% carbohydrate, of which 3% was fiber) and 200 mL of water in order to induce postprandial gastrointestinal motility before ingesting the wireless motility capsule. Participants were instructed not to eat for the following six hours and were supplied with a diary for entry of eating, sleeping, and bowel movements until the capsule was expelled. Data were analysed by a single observer using the commercially available software package MotiliGI (version 3.0.20; Given Imaging). Segmental transit times were estimated based on the elapsed time between physiological pH-landmarks. Prolonged transit times were defined based on normative data [16].

### 2.4. Symptoms of Gastroparesis

Subjective symptoms of gastroparesis were recorded using the Gastroparesis Cardinal Symptom Index questionnaire consisting of nine items covering three subdomains (nausea/vomiting, bloating, and early satiety) [17]. Each item was rated on a 6-point Likert scale ranging from “no symptoms” to “very severe symptoms”. Mean scores for each subdomain as well as an overall mean total symptom score were calculated. 

### 2.5. Statistics

Data distribution was assessed using the Shapiro-Wilks test of normality, and based on this, a one-way ANOVA or the parametric equivalent Kruskal-Wallis was used for comparisons between groups. Post hoc analyses included the Bonferroni correction or Dunn’s multiple comparisons test as appropriate. Sex-related differences in cytokine expression in the diabetes cohorts were analysed using a Mann-Whitney U test. For investigations of associations between serum concentrations of inflammatory cytokines and gastrointestinal transit times, type 1 and type 2 diabetes were pooled, and logistic regressions, including disease duration and HbA1c as confounders, were performed. Associations between inflammatory cytokines and scores of cardinal symptoms of gastroparesis were investigated by Spearman’s rank correlation coefficient. Statistical significance was defined as *p*-values < 0.05 using Stata software (Stata/MP, version 16). 

## 3. Results

### 3.1. Study Population

Participants with type 1 diabetes had longer disease duration (*p* < 0.001) and higher HbA1c (*p* = 0.002) compared to those with type 2. Participants with type 2 diabetes were older (*p* < 0.001) and had higher body mass index (*p* < 0.001) compared to both healthy and people with type 1 diabetes. No differences between groups were seen in the distribution of male and female participants in the diabetes cohorts (Table 1A). Insulin therapies were more frequent in type 1 diabetes compared to type 2, while oral antidiabetics, antihypertensive, and statin therapy were more prevalent in the type 2 diabetes cohort. Regarding diabetic complications, neuropathy was more frequent in type 2 diabetes, while retinopathy was more frequent in type 1. No differences between types were seen for nephropathy and cardiac autonomic neuropathy. 

### 3.2. Inflammatory Cytokines

A total of 19 samples (healthy: 1; type 1 diabetes: 4; type 2 diabetes: 12) were excluded from the dataset due to CRP levels above 10 mg/L indicating acute inflammatory processes. Furthermore, two samples (healthy: 0; type 1 diabetes: 0; type 2 diabetes: 2) were excluded due to haemolysis. In the remaining samples (healthy: 20; type 1 diabetes: 52; type 2 diabetes: 86), inflammatory cytokines were successfully measured in 99% (IL-6, TNF-α) and 100% (IL-8, IL-10, IFN-γ) of cases. In four out of five cytokines (IL-6, IL-8, TNF-α, and IFN-γ), serum concentrations were higher in type 2 diabetes compared to type 1 (*p* < 0.003) (Table 1B). No differences between groups were seen in IL-10 concentrations. Only TNF-α differed between healthy and diabetes cohorts, with increased levels compared to type 1 (*p* = 0.049) and decreased levels compared to type 2 (*p* = 0.008). In type 1 diabetes, levels of IL-6 (*p* = 0.030) and TNF-α (*p* = 0.019) were increased in males compared to females. No sex-related differences were seen in the type 2 cohort

### 3.3. Gastrointestinal Transit Times

Segmental transit times were successfully measured in 94% for gastric emptying, 92% for small bowel transit, and 88% for colonic transit. No differences between type 1 and type 2 diabetes were seen in transit times or the proportion of individuals with prolonged transit times (Table 1C). Contrarily, the diabetes cohorts had significantly longer colonic transit times compared to the healthy controls (*p* < 0.04).

### 3.4. Inflammatory Cytokines and Prolonged Gastrointestinal Transit Times

**Figure 1:**
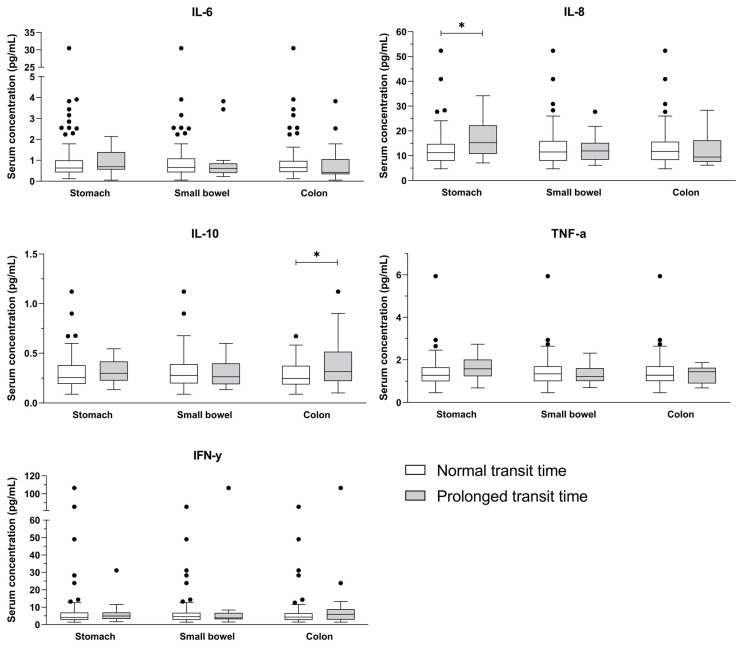
Serum concentrations of cytokines (IL-6, IL-8, IL-10, TNF-α, IFN-γ) in normal and prolonged transit time of the stomach, small bowel, and colon. Outliers are seen as circles. * *p* < 0.05.

Serum concentrations of IL-8 were associated with prolonged gastric emptying time (odds ratio (OR) 1.07, *p* = 0.027), while IL-10 levels were associated with prolonged colonic transit time (OR 29.99, *p* = 0.013) (Figure 1). These results were unaffected by disease duration and glycaemic control (Table 2). No significant differences were observed for IL-6, TNF-α, IFN-γ, or small bowel transit. 

### 3.5. Inflammatory Cytokines and Symptoms of Gastroparesis

Inverse correlations between IL-6 concentrations and nausea/vomiting (rho = −0.19, *p* = 0.026) and bloating (rho = −0.29; *p* < 0.001) were seen (Table 3). No correlations with the remaining cytokines were found. 

## 4. Discussion

In this study, we explored the associations between systemic levels of inflammatory markers and diabetic gastroenteropathy and used segmental transit times as a proxy to assess the health of the enteric nervous system. We showed that prolonged gastric emptying in diabetes was associated with increased serum levels of IL-8, while increased levels of IL-10 were associated with prolonged colonic transit time. Surprisingly, we found that the subjective cardinal symptoms of gastroparesis were inversely associated with levels of IL-6. 

### 4.1. Inflammatory Cytokines and Gastrointestinal Transit Times

Cytokines influence gastrointestinal motility. This has been shown in several preclinical studies, and evidence from human studies indicates a direct mechanism between local cytokine profiles in the gastrointestinal tract and altered motility patterns [18]. For instance, it is well-established that the development of ileus after abdominal surgery is influenced by mechanical activation of macrophages residing in the wall of the gastrointestinal tract causing the release of especially Th1 cytokines leading to impaired motility and inefficient propulsive motions [19]. On the other hand, Th2 cytokines are generally considered motility-inducing, but some discrepancies in reported findings are seen, and the specific effect of a given cytokine on motility may be disease- and patient-specific [18,20]. The exact mechanism by which cytokines affect gastrointestinal motility is poorly understood. However, since cells responsible for peristalsis, including smooth muscle cells, interstitial cells of Cajal, and neurons of the enteric nervous system, all seem to express cytokine receptors [18], it may be plausible to hypothesize that direct interactions between local inflammation and motility exist. 

In diabetes, prolonged segmental gastrointestinal transit contributes to common symptoms of gastroenteropathy, including nausea, early satiety, bloating, and constipation [1]. Due to metabolic alterations, individuals with diabetes are known to have systemic low-grade inflammation [9]. This pro-inflammatory environment induces neurotoxicity, thereby contributing to the development of neuropathies of both peripheral and autonomic nerves [21,22]. The enteric nervous system plays a crucial role in controlling motility, and it seems likely that the pro-inflammatory neurotoxic pathways in diabetes also affect the enteric nerves thereby contributing to gastroenteropathy. There is, however, a substantial knowledge gap regarding this connection. In this study, we investigated systemic levels of cytokines and prolonged gastrointestinal transit as a proxy for enteric nervous system dysfunction. To our knowledge, an association between IL-8 and gastric emptying time in people with diabetes has not previously been reported. However, a study in patients with ulcerative colitis and local inflammation has reported such a relationship between elevated cytokine profiles of IL-8 and prolonged orocecal transit [6], which contrasts in vitro findings showing that IL-8 in a dose-dependently manner increases acetylcholine-induced contractility of ileal and colonic segments [23]. In the gastrointestinal tract, other factors such as pylorospasms may also contribute to the delayed passage of food to the duodenum [24], explaining at least some of the differences between clinical and preclinical findings. 

Surprisingly, we found no difference in circulating IL-6 between individuals with or without prolonged gastric emptying. Acute exercise-induced rises in IL-6 have been shown to delay gastric emptying, thereby improving glycaemic control [25], and drastic IL-6 increase following abdominal surgery correlate with gastric motility [26]. Furthermore, decreased IL-6 secretion through inhibition of M1 polarization of gastric macrophages accelerated gastric emptying in a murine model of delayed gastrointestinal motility [27]. Given that we observed no associations between IL-6 and gastric emptying time, it can be speculated if the subtle levels of IL-6 in our cohorts were insufficient in affecting gastric motility. Likewise unexpectedly, we found no associations between gastrointestinal transit times and TNF-α, even though this cytokine has been associated with prolonged transit time in ulcerative colitis [6], and accumulating preclinical evidence shows that this potent pro-inflammatory cytokine may play an active role in modulation of motility patterns within the gastrointestinal tract. In vitro studies suggest that TNF-α reduces the number of interstitial cells of Cajal and has an inhibitory effect on the contractility of the colonic smooth muscle layer [28,29,30]. Additionally, the administration of a mitogen-activated protein kinase (MAPK) inhibitor in a rat model of diabetic gastroparesis significantly reduced TNF-α concentration in the gastric wall and improved gastric emptying rate [31]. Our results, however, suggest that these observations may not be translational to humans, but verifications in larger cohorts are needed. 

Furthermore, IL-10, which historically has been linked with anti-inflammatory properties [32], was associated with prolonged colonic transit time in our study, supporting similar findings in a cohort of individuals with ulcerative colitis [6]. In line with this, an in vitro study showed that IL-10 was linked to disturbed motility in postoperative ileus, indicating that this anti-inflammatory cytokine has the potential to facilitate peristaltic disturbances [33]. These interesting findings regarding the role of IL-10 on prolonged colonic transit need further elucidation, especially considering the fact that anti-inflammatory cytokines are not commonly considered part of the pathophysiology. In contrast, several studies have shown that the pro-inflammatory cytokine IL-6 has an influencing role in colonic motility. Animal studies have found IL-6 to have a stimulatory effect on colonic contractility [34]. A meta-analysis on IL-6 plasma levels in patients with diarrhoea-predominant irritable bowel syndrome (IBS) revealed that patients had significantly higher IL-6 levels compared to healthy, while no differences were seen between constipation-predominant IBS and healthy [35]. Moreover, elevated levels of IL-6 were associated with prolonged orocecal transit in ulcerative colitis [6]. Based on this, we also expected to see an association between IL-6 and colonic transit time in our cohort. 

The low number of individuals with prolonged gastrointestinal transit time in our cohorts hindered the analysis of an association with low-grade inflammation based on diabetes type. However, in our cohorts, pro-inflammatory cytokines IL-6, IL-8, TNF-α, and IFN-γ were significantly higher in type 2 diabetes compared to type 1, which is consistent with previous findings [36,37,38]. It could be hypothesized that this elevated inflammatory profile would be reflected in a more pronounced dysfunction of the gastrointestinal tract in type 2 diabetes. However, this theory is contradicted by the fact that the risk of developing gastroparesis is known to be substantially higher in type 1 diabetes compared to type 2 (30). This underlines the fact that the pathogenesis is multifactorial and not solely a consequence of pro-inflammatory processes. Additionally, for future studies in larger cohorts, it would be interesting to look into the increased levels of IL-6 and TNF-α in males compared to females in the type 1 cohort and the potential impact on gastrointestinal transit times. 

### 4.2. Inflammatory Cytokines and Symptoms of Gastroparesis

The cardinal symptoms of gastroparesis include nausea, early satiety, and bloating [17]. It is, however, well-described that gastrointestinal symptoms and objective measures of motility are poorly associated. Indeed, gastroparesis may or may not be accompanied by bothersome symptoms in diabetes [5,39]. Perhaps enteric motility is better correlated with symptoms of gastroparesis [40]. In this study, we found an inverse correlation between levels of IL-6 and symptoms of nausea/vomiting and bloating. This suggests that increased levels of IL-6 are protective against these symptoms, which is both counterintuitive and unexpected as IL-6 previously has been linked to prolonged gastric emptying time [25,26,27]. 

### 4.3. Strengths and Limitations

The application of high-tech and precise and reliable methods for the investigation of inflammatory factors and segmental transit times are obvious strengths of this study [41,42]. Thus, our data comprise a unique, high-quality characterization of systemic low-grade inflammation and transit times in a cohort of people with diabetes. Moreover, the medical treatment in our cohort mirrored National guidelines for antidiabetic treatment, and the prevalence of diabetic complications was in line with other studies suggesting generalizability to the diabetes population [43,44]. The study is, however, conducted with limitations. Firstly, data is based on secondary analyses and, as such, are subject to certain limitations, e.g., risk of being underpowered, and therefore, the lack of expected associations between some inflammatory markers and transit times may be the result of type II errors. Thus, larger studies designed with sufficient power for detecting subtle associations between systemic inflammation and gastrointestinal transit times are warranted. Secondly, we measured systemically inflammatory markers instead of locally from biopsies of the gastrointestinal wall. While easier obtainable, systemic inflammatory cytokines are influenced by multiple factors. Nonetheless, our results indicate an association between some inflammatory cytokines and dys-coordinated motility patterns in diabetes. Thirdly, as this is a cross-sectional study, our data do not allow interpretation of whether these factors are linked by causality. To answer that, prospective studies are required in which inflammatory factors are measured both systemically and locally. Fourthly, future studies should also include information regarding factors known to influence gastrointestinal function, such as diet, exercise, level of stress, and gut microbiome. Finally, inclusion was not aimed at people with gastrointestinal problems, and indeed our cohort was largely asymptomatic [5], reflected in the relatively low number of individuals with prolonged segmental transit times. However, the fact that we were able to show statistically significant differences in serum concentrations of inflammatory factors between individuals with normal and prolonged transit times, despite the uneven distribution in the groups, indicates that an association is likely existing and should be investigated in a symptomatic cohort. 

## 5. Conclusions

In this hypothesis-generating study, we found an association between gastrointestinal transit times and systemic inflammatory cytokines. This could indicate that gastroenteropathy in diabetes is influenced by low-grade systemic inflammation associated with long-term disease duration. However, we also found an inverse correlation between pro-inflammatory IL-6 and symptoms of gastroenteropathy. Taken together, the results of this study reveal an inconclusive pattern as to the associations between systemic inflammatory cytokines and measures of gastroenteropathy. Additional knowledge within this field could form the basis for a novel targeted anti-inflammatory therapeutic approach for this complex and debilitating complication in diabetes. 

## Figures and Tables

**Table 1:** Characteristics of cohorts.

	Healthy (*n* = 20)	Type 1 Diabetes (*n* = 52)	Type 2 Diabetes (*n* = 86)
**A: Demography**			
Age (years)	52 (47;56)	45 (28;59)	66 (59;71) **†‡**
Sex (male)	75%	50% **†**	65%
BMI (kg/m^2^)	25.2 (23.3;27.7)	25.8 (23.6;27.8)	30.9 (26.7;33.7) **†‡**
HbA1c (mmol/L)	33 (33;34)	60 (53;70) **†**	55 (48;60) **†‡**
Disease duration (years)	-	21 (13;33)	10 (5;16) **‡**
**B: Medication**			
Insulin	-	100%	22% **‡**
Long-acting	-	62%	22% **‡**
Short-acting	-	63%	6% **‡**
Pump	-	38%	0% **‡**
Oral antidiabetics	-	2%	95% **‡**
Antihypertensiva	-	37%	70% **‡**
Statins	-	33%	73% **‡**
**C: Diabetic complications**			
Neuropathy	-	33%	60% **‡**
Nephropathy	-	16%	17%
Retinopathy	-	37%	6% **‡**
Cardiac autonomic neuropathy	-	33%	40%
**D: Inflammatory cytokines**			
IL-6 (pg/mL)	0.55 (0.44;0.87)	0.47 (0.31;0.70)	0.88 (0.58;1.17) **‡**
IL-8 (pg/mL)	11.64 (10.0;13.90)	10.0 (7.6;11.8)	13.3 (9.4;16.8) **‡**
IL-10 (pg/mL)	0.23 (0.18;0.33)	0.28 (0.19;0.38)	0.27 (0.20;0.39)
TNF-α (pg/mL)	1.27 (1.08;1.40)	1.00 (0.84;1.18) **†**	1.51 (1.27;1.79) **†‡**
IFN-γ (pg/mL)	4.90 (3.58;5.59)	3.45 (2.47;5.68)	5.24 (3.12;7.81) ‡
**E: Transit times**			
Gastric emptying time (min)	203 (178;228)	193 (137;238)	194 (152;239)
GET prolonged (%)	0%	12%	15%
Small bowel transit time (min)	272 (197;335)	280 (243;409)	268 (196;354)
SBTT prolonged (%)	16%	18%	16%
Colonic transit time (min)	984 (720;1549)	1541 (1028;2435) **†**	1512 (975;2593) **†**
CTT prolonged (%)	5%	15%	17%

Demography (A), medication (B), diabetic complications (C), inflammatory cytokines (D), and transit times (E) of participants after exclusion due to C-reactive protein levels > 10 mg/L and haemolysis of the blood samples. Results are displayed as median (1st; 3rd quartiles) or percentages. **†** Significantly different from the healthy cohort (*p* < 0.05), **‡** Significantly different from the type 1 diabetes cohort (*p* < 0.05). BMI: Body mass index, GET: Gastric emptying time, SBTT: Small bowel transit time, CTT: Colonic transit time.

**Table 2:** Associations between gastrointestinal transit times and inflammatory markers.

		Unadjusted Model		Adjusted Model	
		OR (95% CI)	*p*-Value	OR (95% CI)	*p*-Value
**Gastric** **emptying time**	IL-6	0.94 (0.66–1.35)	0.738	0.95 (0.63–1.44)	0.813
	IL-8	1.07 (1.01–1.14)	**0.027**	1.09 (1.0–1.17)	**0.009**
	IL-10	1.85 (0.09–39.81)	0.694	2.17 (0.07–64.89)	0.655
	TNF-α	1.68 (0.85–3.33)	0.134	1.76 (0.88–3.52)	0.111
	IFN-γ	0.99 (0.95–1.04)	0.819	1.00 (0.96–1.05)	0.916
**Small bowel transit time**	IL-6	0.97 (0.79–1.20)	0.812	0.96 (0.79–1.18)	0.712
	IL-8	1.01 (0.95–1.07)	0.868	1.00 (0.94–1.07)	0.895
	IL-10	0.39 (0.02–9.43)	0.559	0.49 (0.02–13.17)	0.669
	TNF-α	0.81 (0.35–1.87)	0.627	0.73 (0.29–1.82)	0.495
	IFN-γ	1.01 (0.98–1.04)	0.450	1.01 (0.98–1.04)	0.609
**Colonic** **transit time**	IL-6	0.98 (0.81–1.18)	0.814	1.00 (0.83–1.20)	0.995
	IL-8	0.10 (0.94–1.06)	0.916	1.00 (0.94–1.06)	0.950
	IL-10	29.99 (2.02–445.18)	**0.013**	26.33 (1.70–408.47)	**0.019**
	TNF-α	0.90 (0.44–1.84)	0.766	0.90 (0.43–1.89)	0.783
	IFN-γ	1.02 (0.99–1.04)	0.274	1.02 (0.99–1.05)	0.158

Odds ratio (OR) for associations between gastrointestinal transit times and serum concentrations of inflammatory factors in diabetes unadjusted and adjusted for disease duration and HbA1c. Boldface font indicates statistical significance (*p* < 0.05).

**Table 3:** Correlations between gastrointestinal symptoms and inflammatory markers.

	Nausea/Vomiting		Early Satiety		Bloating		Total Symptom Score	
	r_s_	*p*-Value	r_s_	*p*-Value	r_s_	*p*-Value	r_s_	*p*-Value
IL-6	−0.19	0.026	−0.07	0.410	−0.29	<0.001	−0.16	0.072
IL-8	−0.16	0.0645	−0.04	0.652	−0.14	0.102	−0.071	0.423
IL-10	−0.07	0.409	0.047	0.598	0.01	0.948	0.02	0.819
TNF-α	−0.14	0.113	0.02	0.824	−0.09	0.310	−0.03	0.759
IFN-γ	−0.13	0.151	0.074	0.404	−0.14	0.099	0.00	0.988

Spearman’s rho (r_s_) and corresponding *p*-value for correlations between inflammatory markers and gastrointestinal symptoms are shown. Bold font indicates statistical significance (*p* < 0.05).

## Data Availability

The data that support the findings of this study are available from the corresponding author upon reasonable request.
